# Statement of the Prolamin Working Group on the Determination of Gluten in Fermented Foods Containing Partially Hydrolyzed Gluten

**DOI:** 10.3389/fnut.2020.626712

**Published:** 2021-01-12

**Authors:** Katharina Anne Scherf, Carlo Catassi, Fernando G. Chirdo, Paul J. Ciclitira, Conleth Francis Feighery, Carmen Gianfrani, Frits Koning, Knut E. A. Lundin, Stefania Masci, Detlef Schuppan, Marinus J. M. Smulders, Olivier Tranquet, Riccardo Troncone, Peter Koehler

**Affiliations:** ^1^Department of Bioactive and Functional Food Chemistry, Institute of Applied Biosciences, Karlsruhe Institute of Technology (KIT), Karlsruhe, Germany; ^2^Department of Pediatrics, Polytechnic University of Marche, Ancona, Italy; ^3^Instituto de Estudios Inmunologicos y Fisiopatologicos, Universidad Nacional de La Plata, La Plata, Argentina; ^4^Faculty of Medicine and Health Sciences, Norwich Medical School, University of East Anglia, Norwich, United Kingdom; ^5^St. James's Hospital, University of Dublin, Dublin, Ireland; ^6^Institute of Biochemistry and Cell Biology, Italian National Council of Research, Naples, Italy; ^7^Department of Immunology, Leiden University Medical Centre, Leiden, Netherlands; ^8^Stiftelsen KG Jebsen Coeliac Disease Research Centre, Department of Gastroenterology, Oslo University Hospital Rikshospitalet, University of Oslo, Oslo, Norway; ^9^Department of Agricultural and Forestry Sciences, University of Tuscia, Viterbo, Italy; ^10^Institute for Translational Medicine, University Medical Center of the Johannes Gutenberg University Mainz, Mainz, Germany; ^11^Plant Breeding, Wageningen University and Research, Wageningen, Netherlands; ^12^INRAE, UR BIA, Nantes, France; ^13^European Laboratory for the Investigation of Food Induced Diseases, Department of Medical Translational Sciences, University Federico II, Naples, Italy; ^14^Biotask AG, Esslingen am Neckar, Germany

**Keywords:** analysis, competitive ELISA, fermented food, gluten, LC-MS/MS, partially hydrolyzed gluten, Prolamin Working Group

On August 12, 2020, the U.S. Food and Drug Administration (FDA) has finalized a rule related to gluten-free labeling for foods containing fermented, hydrolyzed ingredients. The FDA believes that there is no scientifically valid analytical method effective for determining gluten in fermented or hydrolyzed foods. In the absence of an analytical method, the FDA has decided to evaluate gluten-free claims on these foods based only on evidence that the food or ingredient used is gluten-free before fermentation or hydrolysis. For example, barley-based beers from which gluten is removed during brewing using special filtration, adsorption and/or enzymatic treatment are therefore excluded from bearing a gluten-free label.

The Prolamin Working Group (PWG) acknowledges that the FDA rule is a regulatory act and might have to take into consideration several aspects other than scientific evidence, including risk assessment. Nevertheless, the PWG thinks that science has to be the most important driver for regulatory acts in risk management.

In contrast, in the EU such beers are currently allowed to bear a gluten-free label. As required by Regulation (EU) No 1169/2011 of 25 October 2011 on the provision of food information to consumers, the ingredients list must include “barley malt” in highlighted lettering, because gluten-containing cereals are listed in Annex II of the Regulation as substances or products causing allergies or intolerances. The maximum gluten level to bear a gluten-free claim is set in Regulation (EU) No 828/2014 of 30 July 2014 on the requirements for the provision of information to consumers on the absence or reduced presence of gluten in food. On this legal basis, non-governmental Organizations such as the Association of European Coeliac Societies (AOECS) have developed the European Licensing System with guidelines that have to be met to allow using the crossed grain symbol for gluten-free food on the label.

This difference of regulation is a topic of much debate at the moment, because of the divergent opinions between the FDA and the EU. Today, enzyme-linked immunosorbent assays (ELISAs) are the methods of choice for gluten quantitation and are widely used in routine analysis of gluten in food. The R5 antibody, the most prevalent monoclonal antibody used in gluten analysis, was developed by Enrique Mendez, a member of the PWG and is available as a Sandwich ELISA for intact gluten and a competitive ELISA for partially hydrolyzed gluten. The R5 Sandwich ELISA has been endorsed by Codex Alimentarius as a Type 1 method to determine gluten in food. However, the Sandwich format is not suitable to determine gluten in products containing partially hydrolyzed gluten, and competitive ELISAs are required for this type of analysis. Competitive ELISAs based on either R5, G12 or DQ2.5-glia-α3 antibodies are currently available, even if their use is not yet fully approved by official food control authorities. The following statements on the R5 ELISA might be also valid for other competitive ELISAs. Based on the available scientific studies, the PWG thinks that the competitive R5 ELISA is suitable to determine the gluten content of fermented foods containing partially hydrolyzed gluten from wheat, rye or barley, such as beer. The PWG acknowledges that this method has limitations because it might miss smaller peptide fragments and might not have the perfect standard to account for the vast variety of different fermentation procedures common in food processing. However, this method has been validated by an international collaborative study under the guidance of the PWG in 2013. Beer spiked with partially hydrolyzed gluten, naturally gluten-contaminated starch syrup and dried sourdough were used as matrices. The collaborative study was successful and the R5 competitive ELISA was subsequently approved by the respective expert panels as AACCI Method 38-55.01 and AOAC Official Method of Analysis (Final Action OMA 2015.05) because the method was shown to be accurate, precise and specific for its intended purpose. An additional study was initiated with internationally known experts to show the reliability of the R5 competitive ELISA method for the investigation of beer samples. A subcommittee of the American Society of Brewing Chemists recommended that the method for gluten determination by R5 competitive ELISA be included in their Methods of Analysis. The PWG therefore considers that this method is currently state-of-the-art to quantitate gluten in fermented foods.

The FDA thinks that the R5 competitive ELISA method is not suitable for the detection and quantitation of gluten in any fermented or hydrolyzed food because of different hydrolytic conditions in the food to be analyzed and in the material used for calibration. This opinion is scientifically correct but following this reasoning would imply that a different calibrator has to be prepared for each sample matrix, i.e., for each type of beer, even from the same producer. Thus, standardization of the method would be impossible and results would not be comparable between different laboratories or manufacturers. This would be a step back in gluten analysis. Furthermore, the same issue also applies to other methods used for gluten analysis, e.g., sandwich ELISA or liquid chromatography tandem mass spectrometry (LC-MS/MS). In this field of research, calibrators are always a compromise between different possibilities.

The most important alternative method to detect gluten in fermented foods is LC-MS/MS. This technique is currently able to detect and quantify gluten fragments (peptides) in beers but so far there is no validated routine method to give absolute values for the gluten content based on these fragments. The PWG has not seen any values in mg/kg of a CD-active peptide that would really allow to make a good statement if these traces would be relevant to CD patients or if the contents are so low that they fall below the 20 mg/kg threshold for gluten-free foods. Even if modern MS equipment is able to detect femtomolar amounts of peptides, a proper risk assessment of the detected contents is not available.

It is often said that the R5 competitive ELISA does not provide information on the immunogenicity of the detected fragments. However, this is also true for alternative analytical methods for gluten quantitation, and, in addition, this is not the intended use of the method. Food analytical methods can only determine the content of an analyte and are relevant for the decision if this analyte is below or above a threshold set by legislation. Immunogenicity can only be evaluated in clinical studies.

The PWG thinks that more scientific studies are needed to (i) better understand MS-detection of residual gluten in beer and other fermented foods and also (ii) to ensure that the R5 competitive ELISA picks up each peptide fragment it should. Also, MS detection and quantitation of gluten fragments is not feasible for food manufacturers, in particular small companies, because it is too expensive and too demanding in terms of time and skill of the operators. Thus, it is difficult to recommend it as a routine testing method also having in mind that a proper method for absolute gluten quantitation by MS is not available so far.

## Author Contributions

KS and PK wrote the first draft of the manuscript. All authors contributed to revising and editing the manuscript and approved the final manuscript.

## Conflict of Interest

The authors declare that the research was conducted in the absence of any commercial or financial relationships that could be construed as a potential conflict of interest.

